# Investigating the Factors Behind Patients' Desire and Decision to be Accompanied: A Cross‐Sectional Analysis

**DOI:** 10.1111/hex.70102

**Published:** 2025-01-05

**Authors:** Mehmet Göktuğ Kılınçarslan, Büşra Dönmez, Yasemin Kaya Beştepe, Büşra Nur Kırıkcıoğlu, Merve Akbaşoğlu, Bezar Karakaya, Erkan Melih Şahin

**Affiliations:** ^1^ Department of Family Medicine, Faculty of Medicine Çanakkale Onsekiz Mart University Çanakkale Turkey

**Keywords:** anxiety, companions, developing countries, health literacy, social support

## Abstract

**Introduction:**

Medical education typically focuses on the dyadic interaction between patient and physician. However, there is another significant presence in the room that can also impact the patient's health outcomes: caregivers. This topic has been relatively underexplored until now, and there is insufficient information available regarding situations in different cultures. In this study, we aimed to separately examine the characteristics of patients that influence the frequency of being accompanied and those that affect patients' preferences regarding the presence of a companion.

**Methods:**

This cross‐sectional study was conducted in family medicine clinic of a tertiary hospital. During a period of 15 days, a total of 285 patients who visited the clinic were administered the questionnaire face‐to‐face. Two logistic regression models were used for dependent variables of “actual” and “desired” situations of admitting to healthcare service with companion.

**Results:**

Of the participants, 167 (58.6%) were female, and the mean age was 36.8 ± 16.2 The sole significant factor, influencing actual visits to be occurred with a companion, was the solution for transportation issues (odds ratio [OR]: 26.25). It was found that unmarried individuals (single/divorced/widowed) (OR: 5.47), those with higher income (OR: 1.84), and older individuals (OR: 1.04) had a higher tendency to prefer visiting the clinic with companion while female are as opposite (OR: 0.50). Anxiety, perceived social support, and health literacy weren't associated with actual situation or desire to have companion.

**Conclusion:**

Patients have companions to address tangible issues. However, different factors may influence the desire to have a companion. There is a large group of individuals who, are accompanied at clinic visits against their wishes, indicating a conflict between being accompanied and the desire for one.

**Patient or Public Contribution:**

Our study was inspired by the unsolicited comments of patients made about their companions during clinical visits. Additionally, community provided valuable feedback during the pilot application phase, particularly in the development of the data form.

## 1 Introduction

Traditionally, medical education focuses on the encounter between two individuals: the patient and the physician. In fact, previous research has routinely excluded triadic consultations from analysis, labelling them as 'contaminated'. [1]. However, in practice, a third person often accompanies the patient during medical encounters [2]. In adult consultations, a companion is present in approximately one‐third to half of the cases [3, 4]. Patients seek information from healthcare providers about the causes of their condition, its expected course, and treatment options, benefits, side effects, and other uncertainties. Information sharing is particularly challenging for patients with complex physical symptoms, psychosocial stressors, and medically complex conditions, and these patients tend to have companion [3].

Companions not only support instrumental and task needs but also provide relational support [5]. The accompanying individual can offer important insights into the patient's psychological and socio‐cultural dimensions [6]. Having a companion during clinical visits plays a crucial role in communication between the patient and the family physician [7]. Companions are more than mere accessories; they are part of the patient‐physician communication. Rather than constraining, they enhance the patient–physician interaction [8]. They may facilitate or hinder the patient's participation and autonomy in decision‐making [9]. Even if they do not actively contribute, the patient is given the opportunity to discuss their condition with someone from their own social circle, which can provide valuable insights for the doctor witnessing the conversation [10].

The presence of a companion can impact key patient outcomes, such as the patient's understanding, the physician's comprehension, the number of requested tests, prescriptions, and referrals [6]. Evidence suggests that the more actively involved family members are in medical visits, the greater the patient satisfaction with their usual care provider and the more actively patients participate in decision‐making [7]. Many authors argue that triadic participation shows great promise for cost‐effective healthcare delivery [1]. While it may be assumed that the presence of a companion would not negatively affect communication between the patient and the physician, some studies report contrary results [11]. A study of cancer patients demonstrated that companions often spoke on behalf of patients during discussions of prognosis and treatment options, even when the patient was present and capable of self‐advocacy [12]. In a study evaluating pre‐surgical triadic consultations, the presence of companions altered patient‐surgeon communication by increasing the medical focus of the discussion, with surgeons conveying more medical information and patients disclosing less lifestyle and psychosocial information [13]. Interestingly, the same study found no significant differences in post‐visit satisfaction for either patients or surgeons, regardless of whether companions were present [13]. In emergency department settings, the presence of a companion has been associated with adverse outcomes, such as delays in diagnosis [14]. A comprehensive review of triadic consultations reveals conflicting findings across a wide range of studies [3].

The ambiguity surrounding the outcomes of visiting with a companion becomes more pronounced when considering the characteristics of accompanied patients. While some studies have demonstrated that female gender [15] or male gender [16] is associated with an increased likelihood of visiting with a companion, other studies [6, 17] report no significant relationship with gender [18]. Similar to gender, the influence of other factors such as age, education level, overall health, and mental health on the frequency of visiting with a companion has also yielded varying results. [3, 19]. Meta‐analyses generally show that female gender, lower education level, and poor physical health are associated with an increased likelihood of visiting with a companion. However, studies examining scenarios such as oncological visits, where demographic factors may not be as influential, have reported differing outcomes [3, 19]. The situation is similar with regard to mental health. While some studies have found no association between mental health status and the frequency of being accompanied [20, 21], other research [17] has identified a link between depressive symptoms and increased likelihood of being accompanied. These three studies were conducted on different groups, such as elderly patients, breast cancer patients, and those with heart failure and diabetes. In a study by Del Piccolo et al. [21] investigating breast cancer patients and the frequency of visiting with a companion in relation to mental health, no significant relationship was found, which was attributed to the already high levels of anxiety and depressive symptoms in this population.

Another characteristic thought to influence the frequency of being accompanied is low health literacy, and studies have confirmed the existence of this association [17, 22]. Social support has also been proposed as a factor affecting the frequency of visiting with a companion. In the literature, only two studies have examined this aspect through family functionality, and both observed that good family relationships were associated with increased frequency of visiting with a companion [16, 17].

As can be seen, the literature contains relatively few studies investigating the relationship between patient characteristics and the frequency of visiting with a companion. These relationships have generally been explored using univariate analyses. Moreover, as a recent article highlighted, there is a need for studies that not only examine individual demographic characteristics of the patient and/or companion but also investigate how combinations of these characteristics affect the likelihood of being accompanied [1].

The most recent meta‐analysis on companion visits noted the absence of studies examining the characteristics of patients who do not desire accompaniment [19]. Regardless of cultural differences, there are patients who do not wish for family members to participate in physician consultations, just as there are those who do [23]. An older study reported that no one explicitly stated they never wanted a companion, although about a quarter of patients who were unaccompanied said they would have preferred having one [24]. While it is intriguing that no one in the study expressed a desire to never have a companion, as noted in the limitations of the study, the specificity of the sample may have contributed to this result. It has been demonstrated that in triadic consultations, patient preferences are crucial, and patients who wish to make decisions together with their family tend to experience better outcomes when they were accompanied by family member [25].

Identifying patient characteristics that influence the real and desired conditions of being accompanied will create opportunities to prevent adverse situations and guide future research. In this context, the present study aims to separately examine the characteristics of patients that influence the frequency of being accompanied and those that affect patients' preferences regarding the presence of a companion.

## 2 Materials and Methods

### 2.1 Design and Sample

This cross‐sectional study involved 285 patients who attended the Family Medicine Outpatient Clinic in Turkey between 1 August and 15 August 2023. A total of 303 individuals were reached, but 18 participants declined to participate, resulting in the study being conducted with a total of 285 participants. Patients aged 18 and above were included in the study, while those with physical and/or mental illnesses that would prevent them from responding to the survey were excluded from the study.

In the calculation of the sample size required for logistic regression, the sample size for the small group is 10 times the number of independent variables. In our study, since it is planned to have a maximum of 13 independent variables in logistic regression models, it is aimed to reach a minimum of 260 people in total, with the smallest group being 13 × 10 = 130 [26]. Although we achieved a sample size deemed sufficient by the literature, post hoc power analyses were conducted for both logistic regression models to assess the risk of Type II error. Since post hoc power analyses using observed values can be misleading, the power was recalculated using simulation in R 4.3.3 with the ‘simr’ package [27], assuming a 20% reduction in the model coefficients [28]. In the logistic regression model examining the likelihood of attending with a companion, the power remained at 100% (confidence interval [CI]: 99%–100%) despite a 20% reduction in the coefficients. In the model investigating the desire to be accompanied, the power was 87% (CI: 85%–89%). Larger sample sizes would be required to detect smaller effects; thus, the fact that sufficient power was reached in the models even with smaller effect sizes strengthens the conclusion that our sample size was adequate.

### 2.2 Dependent and Independent Variables

#### 2.2.1 Dependent Variables

Two separate dependent variables were defined for two separate logistic regression:

1. The responses to the question ‘How many people came with you to the clinic?’ were categorised as 0 for those who answered ‘0’ and as 1 for those who answered, ‘1 or higher’, and utilised as dependent variables for the analysis of the actual frequency of patients who have companion.
2. The responses to the question ‘Would you have preferred to come to the clinic with someone?’ were categorised as ‘yes’ and ‘no’ and used as dependent variables for the analysis of the desire for having a companion.

#### 2.2.2 Independent Variables

To determine socio‐demographic characteristics, variables such as age, gender, marital status, education level, place of residence, and income were utilised. Marital status was categorised into married and others. Education level was determined using a Likert‐type scale ranging from 'illiterate' to 'postgraduate degree or above'. Place of residence was recoded as a dichotomous variable, 'city centre” and 'other'. Additionally, the need for assistance from others for transportation, indicated by 'yes' and 'no' responses, was used as an independent variable to evaluate whether transportation needs affected the accompanying to patients. Income status was measured with a 5‐point Likert‐type scale ranging from 'very bad' to 'very good' and used as a continuous variable.

To identify the participants' health conditions that could affect their frequency and style of clinic visits, variables such as the presence of chronic diseases, regular medication use, and the total number of clinic visits in the last 6 months were used as separate independent variables. In the study, the Beck Anxiety Scale, Multidimensional Scale of Perceived Social Support, and Health Literacy Scale‐Short Form were utilised. The Beck Anxiety Scale consists of a total of 21 items rated on a 4‐point Likert scale, with a Cronbach's alpha coefficient of 0.94 [29]. The Multidimensional Scale of Perceived Social Support consists of 12 items rated on a 7‐point Likert scale, with a score range of 12–84. Higher scores indicate higher perceived social support. The scale was developed by Zimet et al. [30], and its validity and reliability study in Turkish were conducted by Eker et al. [31] Turkish version has the Cronbach's Alpha reliability coefficient was found to be 0.93. The Health Literacy Scale‐Short Form consists of 12 items rated on a 4‐point Likert scale, with a score range of 0–50. Higher scores indicate better health literacy. The Cronbach's Alpha reliability coefficient of the scale was found to be 0.856 [32].

### 2.3 Statistical Analysis

Categorical data were presented as frequency (%) while continuous variables were presented as mean ± standard deviation if the data were normally distributed, and as median (1st quartile–3rd quartile) if the data were not normally distributed. Two separate logistic regression models were used for statistical analysis. In the first model, the relationship between having companion and independent variables was examined, while in the second model, the relationship between the desire to have companion and independent variables was examined. Logistic regressions were performed using IBM SPSS v26 software. Odds ratios in the article are reported with 95% CIs. To assess the logistic regressions for multicollinearity, Variance Inflation Factors (VIFs) were calculated using the ‘car’ package in R 4.3.3 [33]. VIF values below 10 do not pose a problem for regression analysis [34].

A significance level of *p* < 0.05 was considered statistically significant.

## 3 Results

Of the participants, 167 (58.6%) were female, and the mean age was 36.8 ± 16.2. The most common educational level among participants was university graduate, with 154 (54.0%) individuals. Details of socio‐demographic and other independent variables are provided in Table 1.

**Table 1:** Characteristics of participants.

Variable	Descriptive statistics
Age	36.8 ± 16.2
Gender	
Male	118 (41.4%)
Female	167 (58.6%)
Marital status	
Married	130 (45.6%)
Single/divorced/widowed	155 (54.4%)
Education Level	
Illiterate	1 (0.4%)
Primary education	50 (17.5%)
High school	44 (15.4%)
University	154 (54.0%)
Postgraduate or above	36 (12.6%)
Place of residence	
City centre	223 (78.2%)
Other	62 (21.8%)
Need for assistance with transportation	
Yes	67 (23.5%)
No	218 (76.5%)
Chronic disease	
Yes	94 (33.0%)
No	191 (67.0%)
Regular medication use	
Yes	112 (39.3%)
No	173 (60.7%)
Frequency of hospital visits in the last 6 months	2 (1–3)
Beck Anxiety Scale Score	8 (4–19)
Multidimensional Scale of Perceived Social Support Score	70 (59–80)
Health Literacy Scale Score	33.8 ± 8.5

Of the participants, 145 (50.9%) visited the family medicine clinic alone during their current visits, while 140 (49.1%) visited with a companion. A logistic regression model was established to examine the relationship between visiting with a companion and independent variables. Our model was statistically significantly better than the null model (*χ* ^2^:86.111. *p* < 0.001), explaining between 26.1%–34.8% (Cox and Snell *R* ^2^: 0.261 ‐ Nagelkerke *R* ^2^:0.348) of the variance. Hosmer–Lemeshow statistic indicates a good fit (*χ* ^2^:9.280, *p*:0.319). It was found that age, gender, marital status, education level, place of residence, income status, presence of chronic illness, continuous medication use, frequency of hospital visits, health literacy, perceived social support and anxiety levels did not affect the admitting with a companion. Only the need for support for transportation significantly increased the frequency of visiting with a companion (OR: 26.25, 95% CI: 9.67–71.29). The logistic regression model is presented in detail in Table 2.

**Table 2:** Logistic regression analysis for clinical visit with companion.

	Coefficient	Odds ratio	95% Confidence intervals		*p*‐Value	VIF
			Lower	Upper		
Age	0.023	1.02	0.99	1.05	0.134	2.71
Gender (male)	0.139	1.15	0.65	2.03	0.632	1.09
Marital status (married)	0.400	1.49	0.70	3.17	0.297	1.90
Place of residence (city centre)	−0.325	0.72	0.33	1.57	0.411	1.18
Education level	−0.026	0.97	0.69	1.37	0.882	1.35
Income	0.264	1.30	0.83	2.03	0.247	1.16
Need for assistance with transportation (No)	3.268	26.25	9.67	71.29	<0.001*	1.08
Cronical disease (No)	−0.774	0.46	0.19	1.12	0.088	2.22
Continuous medication use (No)	0.059	1.06	0.47	2.42	0.888	2.13
Frequency of hospital visits in the last 6 months	−0.007	0.99	0.88	1.12	0.911	1.18
Health Literacy Scale Score	−0.021	0.98	0.94	1.01	0.233	1.22
Perceived Social Support Score	0.017	1.02	1.00	1.04	0.100	1.22
Beck Anxiety Scale Score	0.010	1.01	0.98	1.04	0.468	1.36

*Note:* Cox and Snell *R* ^2^: 0.261 ‐ Nagelkerke *R* ^2^: 0.348. Hosmer–Lemeshow‐*χ* ^2^: 9.280, *p*: 0.319. Along with categorical variables, reference groups have been provided.

Abbreviation: VIF = variance inflation factor.

* *p* < 0.05

*χ* ^2^: 86.111. *p* < 0.001.

Nearly three‐quarters of the participants (210%–73.7%) expressed a preference not to have a companion during their visits to the family medicine clinic, while one quarters (75%–26.3%) indicated a desire for visiting with a companion. A logistic regression model was constructed to examine the relationship between the desire to visit the clinic with a companion and independent variables. Our model was statistically significantly better than the null model (*χ* ^2^: 40.272, *p* < 0.001), explaining between 13.2% and 19.3% (Cox and Snell *R* ^2^: 0.132 ‐ Nagelkerke *R* ^2^: 0.193) of the variance. Hosmer‐Lemeshow statistic indicates a good fit (*χ* ^2^:7.111, *p*:0.524). It was found that unmarried individuals (single/divorced/widowed) (OR: 5.47, 95% CI: 2.27–13.18), those with higher income levels (OR: 1.84, 95 CI%: 1.12–3.02), and older individuals (OR: 1.04, 95% CI: 1.01–1.08) were more likely to express a desire to visit the family medicine clinic with a companion while female are as opposite (OR: 0.50, CI: 0.27–0.93). However, no significant relationship was observed between education level, place of residence, presence of chronic illness, continuous medication use, frequency of hospital visits, health literacy, perceived social support, anxiety levels and the desire to visit the clinic with a companion. However, the relationship between anxiety level and desire for companionship was just slightly above the threshold of significance (*p* = 0.053). All details of the model are provided in Table 3.

**Table 3:** Logistic regression analysis for desire for accompanied visits.

	Coefficient	Odds ratio	95% Confidence intervals		*p*‐Value	VIF
			Lower	Upper		
Age	0.044	1.04	1.01	1.08	0.007*	3.50
Gender (male)	−0.696	0.50	0.27	0.93	0.029*	1.08
Marital Status(married)	1.699	5.47	2.27	13.18	<0.001*	2.31
Place of residence (city centre)	0.267	1.31	0.63	2.70	0.471	1.21
Education level	−0.271	0.76	0.53	1.10	0.146	1.58
Income status	0.610	1.84	1.12	3.02	0.016*	1.21
Chronic disease (No)	−0.059	0.94	0.40	2.22	0.892	2.00
Continuous medication use (No)	−0.265	0.77	0.32	1.84	0.552	2.25
Frequency of hospital visits in the last 6 months	0.069	1.07	0.96	1.19	0.201	1.12
Health Literacy Scale Score	−0.019	0.98	0.94	1.02	0.336	1.21
Perceived Social Support Score	0.016	1.02	0.99	1.04	0.134	1.23
Beck Anxiety Scale Score	0.024	1.02	1.00	1.05	0.053	1.32

*Note:* Cox and Snell *R* ^2^: 0.132 ‐ Nagelkerke *R* ^2^: 0.193. Hosmer–Lemeshow‐*χ* ^2^: 7.111, *p*: 0.524. Along with categorical variables, reference groups have been provided.

Abbreviation: VIF = variance inflation factor.

* *p* < 0.05.

*χ* ^2^: 40.272. *p* < 0.001.

Among the 140 patients who were accompanied, 82 (58.6%) stated that they did not want the presence of a companion, while 17 (11.7%) of the 145 patients who attended alone expressed a desire for a companion. (*χ* ^2^:32.412, *p* < 0.001).

## 4 Discussion

This study revealed being accompanied at clinic visits was significantly associated only with transportation need. After adjusting for other independent variables, people who need transport assistance were 26 times more likely to be accompanied than people who do not need transport assistance. Being accompanied was found to be not associated with other sociodemographic characteristics, anxiety, health literacy, or perceived social support levels.

Secondly, when examined in the context of the desire to be accompanied at visits to family medicine clinics, male gender, being single, having a higher income, and older age were associated with an increased desire to be accompanied. The highest odds ratio, 5.47, was observed for marital status, indicating that single individuals were 5.47 times more likely to prefer to be accompanied compared to those who were married. In contrast, the lowest odds ratio, 0.50, was associated with gender, suggesting that women were half as likely as men to prefer to be accompanied. Additionally, the odds ratio for age was found to be 1.04, meaning that with each year of increased age, individuals were 1.04 times more likely to desire to be accompanied. It is worth noting that this effect grows more pronounced as age differences increase. However, perceived social support, health literacy, and anxiety levels were found to have no influence on the desire for being accompanied.

In the literature, it has been shown that the rate of clinic visits with companion accounts for approximately one‐third to half of all visits [3, 4]. In this study, the rate of clinic visits with companion was nearly half of the total visits. The significantly higher frequency of clinic visits with companion in our country compared to the literature can be attributed primarily to cultural norms and family structure. Other studies reporting ratios in the literature are generally conducted in Western countries, where cultural norms and family structures differ from those in our country [19]. It is possible to say that Eastern families are more collectivist [35]. We would like to emphasise that this could explain the higher rate of companions at clinic visits, observed in our study.

In a meta‐analysis, it was found that older adults generally have family companions during routine medical visits. Patients who have companions are typically older and less educated than patients who attend appointments without companions, and they tend to have more physical and mental health needs [3]. Marriage, low health literacy, and low educational levels have been shown to contribute to increased rate of clinical visits with companion [17, 24]. While studies regarding gender have reported varied results, there are more studies suggesting no difference between genders [18]. Assisting with transportation is consistently reported in the literature as a primary role undertaken by companions [9, 19]. In our study, the effect of gender, marital status, age, education, anxiety, health literacy, or perceived social support levels on being accompanied at clinic visits was not found to be statistically significant. We found that the only factor influencing the frequency of being accompanied was the need for someone to address transportation needs. Different results are observed in various societies and cultures regarding clinic visits with a companion. For example, in our study, it may have multiple reasons for only transportation needs affecting visits with a companion, while other factors appear to be ineffective when considered together. Firstly, transportation is problematic issue in our country. For instance, according to the United Nations Economic Commission for Europe, as of 2022, the number of passenger cars per capita in our country is nearly one‐fifth of that in the United States and one‐third of that in the UK [36]. Furthermore, the situation is even worse in public transportation. Comparing Germany and Turkey, two countries with similar populations, in 2021, the number of passengers transported by rail in Germany is approximately seven times higher than in Turkey [37]. Considering Maslow's hierarchy of needs, we can see that individuals' physiological and safety needs, such as materialistic needs, should be met first [38]. In this context, it can be said that the intangible factors investigated in our study, such as anxiety, health literacy, and perceptions of social support, may be ranked after physiological needs such as transportation needs in the hierarchy of needs. Due to the unmet nature of basic needs such as transportation, which are prioritised at the top of the hierarchy of needs, in our country, we believe that intangible factors such as anxiety, health literacy, and perceptions of social support do not have a significant impact on frequency of being accompanied.

When examining factors influencing the desire for having a companion, anxiety, health literacy, and perceived social support levels still appear to be ineffective. However, male gender, unmarried status, having a good income, and older age were found to increase the desire for having a companion. It is particularly noteworthy that the increased desire for having a companion among the unmarried participants. Study conducted in most Western culture has found that being married increases the frequency of attending with a companion [24]. The finding in our study, which shows that unmarried individuals express a greater desire for a companion, may seem contradictory. However, the study in the literature was conducted in Western culture and assess the actual situation, without addressing patients' desires. It seems logical for unmarried individuals to seek someone for support in nonroutine actions such as clinic visits, possibly due to feeling lonelier. In this regard, the lack of impact of perceived social support on both actual and desired situations of having a companion in our study raises questions. However, just as majority of the positive effects of being married on physical health operate through perceived social support, the influence of marital status on the desire for companion may also operate through perceived social support [39]. In other words, marital status might have accounted for a significant portion of the variance of perceived social support in predicting desire of companion, leading to its insignificance in the statistical analyses. Further analysis of this situation requires advanced statistical methods. While the effect of anxiety on having a companion is not significant, it is just above the threshold. With a slightly larger sample size, it is likely that the relationship will become statistically significant. Therefore, it is partly feasible to speak of a relationship between high anxiety levels and the desire for having a companion.

Among the 140 patients who were accompanied, 82 (58.6%) stated that they did not want the presence of a companion, while 17 (11.7%) of the 145 patients who attended alone expressed a desire for a companion. As shown in our study, not being accompanied when desired, or even when not desired, being accompanied at clinic visits due to compelling physical reasons, such as the need for transport, is a common situation. Research has shown that in triadic consultations, patient preferences play a key role, and those who prefer to make decisions with their family generally achieve better outcomes when accompanied by a family member [25]. We believe that the findings of our study, particularly regarding patients who had a companion despite not requesting one and those who desired a companion but did not have one, may serve as a basis for future research due to their potential association with worse outcomes.

Since our study sample consisted solely of patients from a single clinic, results should be generalised cautiously to the population. The variance explained in the logistic regression model for the desire to be accompanied was found to be low, indicating that some factors that were not measured in our study may still have a significant effect on this situation. Using techniques such as path analysis or mediation analysis instead of logistic regression could have provided clearer insights into the factors affecting the presence of a companion.

The presence of a companion, which can potentially affect patients' health outcomes [6] and is highly suitable for intervention studies, has been underexplored until now. In our study, as recommended in the literature, the factors affecting patients to be accompanied were evaluated in combination with logistic regression [1]. In developing countries, in accordance with the hierarchy of needs, transportation‐related needs have been identified as the only significant factor influencing the frequency of being accompanied. The most recent meta‐analysis on the subject reported that no studies have assessed the desire for accompaniment by a companion [19]. In this study, it was observed that older individuals, those who are single, and those with higher incomes were less likely to attend with a companion. Moreover, as demonstrated in our study, there is a conflict between the desired and actual situation of being accompanied at clinic visits, which may inevitably lead to negative outcomes [25]. Therefore, identifying the factors that influence the frequency of being accompanied, as well as those that affect the desire for accompaniment, and addressing the conflict between these two situations, could provide valuable guidance for policymakers in implementing measures to resolve this issue.

## 5 Conlusion

This study identified transportation assistance as the sole significant factor influencing the frequency of being accompanied at clinic visits, while sociodemographic characteristics, health literacy, anxiety, and perceived social support levels did not show a significant effect. Additionally, the findings revealed that despite a large proportion of patients being accompanied, many did not express a desire for a companion, indicating a conflict between actual and desired situations. This mismatch between patient preferences and real‐life circumstances, particularly driven by logistical challenges such as transportation, may have potential negative consequences on patient outcomes.

Policymakers should develop strategies aimed at supporting patients who require transportation assistance, contributing to resolving the mismatch between the desire for and the actual presence of a companion at clinic visits. Addressing this discord could help ensure that patient preferences are met and improve overall healthcare experiences. The findings from our study, highlighting the significant presence of companions in at least half of clinical visits, offer valuable insights for future research. Companions can play a crucial role in patient‐physician communication and health outcomes, and our results may inspire further investigation into how best to integrate companions into healthcare practices to optimise patient care.

## 6 Ethics Approval and Informed Consent

Ethical approval for our study was obtained from the Clinical Research Ethics Committee of Canakkale Onsekiz Mart University with the number of 2023/09‐12 on 21/06/2023. All participants provided informed consent before participation in the study. Data were collected anonymously, and no information that could directly identify individuals (e.g., ID) was gathered. Due to the presence of individuals with low health literacy among the participants, the ‘teach‐back’ method was employed during the process of obtaining informed consent from all participants. This method involved receiving feedback from the participant to ensure they understood the steps of the study.

## Author Contributions

**Mehmet Göktuğ Kılınçarslan:** conceptualisation, writing–original draft, methodology, writing–review and editing, formal analysis, data curation, investigation, supervision. **Büşra Dönmez:** conceptualisation, writing–original draft, investigation, writing–review and editing, methodology. **Yasemin Kaya Beştepe:** investigation, writing–review and editing, project administration, methodology, data curation. **Büşra Nur Kırıkcıoğlu:** conceptualisation, software, formal analysis, data curation, writing–review and editing, methodology. **Merve Akbaşoğlu:** data curation, software, resources, conceptualisation, methodology, validation. **Bezar Karakaya:** software, formal analysis, data curation, visualisation, writing–review and editing, conceptualisation. **Erkan Melih Şahi̇n:** supervision, project administration, writing–review and editing, conceptualisation, methodology, data curation.

## Ethics Statement

Ethical approval for our study was obtained from the Clinical Research Ethics Committee of Canakkale Onsekiz Mart University with the number of 2023/09‐12 on 21/06/2023.

## Consent

All participants provided informed consent prior to participation in the study.

## Conflicts of Interest

The authors declare no conflicts of interest.

## Data Availability

The data that support the findings of this study are available on request from the corresponding author. The data are not publicly available due to privacy or ethical restrictions.
